# Association between cognitive deficits and negative symptoms: a systematic review of the literature

**DOI:** 10.1192/j.eurpsy.2023.2220

**Published:** 2023-07-19

**Authors:** A. Melillo, G. M. Giordano, E. Caporusso, F. Tomassini, A. Perrottelli, L. Giuliani, P. Pezzella, N. Sansone, A. Mucci, S. Galderisi

**Affiliations:** Department of Psychiatry, University of Campania “Luigi Vanvitelli”, Naples, Italy

## Abstract

**Introduction:**

In patients with schizophrenia, numerous studies have shown a relationship between negative symptoms and cognitive deficits (both neurocognition and social cognition deficits) and a similar impact of these domains on different clinical features such as onset, course and prognostic relevance. However, this relationship is still today subject of scientific debate.

**Objectives:**

The aim of the present study is to conduct a systematic review of the literature on data concerning the relationships between neurocognition and social cognition deficits and the two different domains of negative symptoms – avolition-apathy and expressive deficit.

**Methods:**

A systematic review of the literature was carried out following PRISMA guidelines and examining articles in English published in the last fifteen years (2007 - March 2022) using three different databases (Pubmed, Scopus and PsychINFO). The included studies involved subjects with one of the following diagnoses: high risk of psychosis, first episode of psychosis, or chronic schizophrenia. Other inclusion criteria of the reviewed studies included: evaluation of at least one neurocognitive or social cognition domain and at least one negative symptom using standardized scales; analysis of the relationship between at least one neurocognitive or social cognition domain and a negative symptom.

**Results:**

Databases search produced 8497 results. After title and abstract screening, 395 articles were selected, of which 103 met inclusion criteria. The analysis of retrieved data is still ongoing. Preliminary evidence highlighted: a correlation between social cognition and negative symptoms, in particular with the “expressive deficit” domain; a positive correlation between the severity of negative symptoms and that of neurocognitive deficits (in particular with the “processing speed” domain); an association of verbal working memory deficits with alogia and anhedonia.

**Conclusions:**

The study of the relationship between negative symptoms, neurocognitive deficits and social cognition could contribute to the understanding of the aetiology of psychotic disorders and therefore to the identification of therapies for the improvement of overall functioning and quality of life. The studies analysed so far show some interesting associations between cognition and negative symptoms, but the presence of often inconsistent results, partially attributable to the different conceptualizations of the various domains of negative symptoms adopted, hinders the generalization of the results.

**Disclosure of Interest:**

None Declared

